# Assessment of Different Technologies in the Fabrication of 3D Printed Tooth Models for Dental Education

**DOI:** 10.1111/eje.70105

**Published:** 2026-02-11

**Authors:** Samuel Bereza, Danilo Carluccio, James Novak, Darius Khadembaschi, Martin Batstone, Saso Ivanovski

**Affiliations:** ^1^ School of Medicine University of Queensland Brisbane Australia; ^2^ School of Dentistry University of Queensland Brisbane Australia; ^3^ Department of Oral and Maxillofacial Surgery Royal Brisbane and Women's Hospital Brisbane Australia; ^4^ Herston Biofabrication Institute, Metro North Health Brisbane Australia; ^5^ School of Architecture, Design and Planning, Faculty of Engineering, Architecture and Information Technology The University of Queensland Brisbane Australia; ^6^ Department of Oral and Maxillofacial Surgery Fiona Stanley Hospital Perth Australia

**Keywords:** access cavity preparation, additive manufacturing, pre‐clinical training, restorative dentistry, typodont

## Abstract

**Background:**

3D printing has emerged as a practical method for fabricating customisable, accurate and affordable dental training models. Numerous 3D printing technologies exist, though there is no standardised comparison to inform their implementation in dental education. This study aimed to evaluate and compare the qualities of various 3D printed dental models against the current standard model used in tertiary dental education.

**Methodology:**

Five maxillary molar tooth models were fabricated using selective laser sintering (SLS), fused filament fabrication (FFF), material jetting (MJ), stereolithography (SLA) and digital light processing (DLP). A standard Ivorine tooth model (One Dental, Australia) was also evaluated. Eighteen participants performed cavity preparations and assessed each model based on haptic feedback, visual appearance, anatomical accuracy and overall suitability using a 5‐point Likert scale.

**Results:**

FFF was the most economical per unit ($0.06), followed by DLP ($0.30). MJ was the most expensive ($1.87), though it offers high resolution and complexity. In terms of enamel haptics and dentine haptics, MJ (3.11, 3.28), SLA (3.00, 3.33) and DLP (3.11, 3.44) were assessed as comparable to Ivorine (3.67, 3.44). Similarly, overall suitability of MJ (3.33), SLA (3.33) and DLP (3.44) was comparable to Ivorine (4.17). FFF and SLS were rated significantly inferior to Ivorine in all domains.

**Conclusions:**

Current 3D printed models do not fully simulate dental tissue and should be used to supplement the limitations of standard models and improve self‐learning. Future research is warranted to develop models with improved haptic feedback and realistic surface anatomy.

## 1 Introduction

The use of tooth models to simulate dental procedures is important in dental education, allowing students to hone their technical skills in a standardised environment without the risk of causing iatrogenic harm to patients. Extracted human teeth have historically been used in preclinical dental education to facilitate the practice of a variety of dental procedures with a high degree of fidelity in relation to natural tooth anatomy and haptic feedback [1]. In recent years, especially after the COVID‐19 pandemic, extracted teeth have fallen out of favour due to the potential risk of infection transmission, as well as ethical and practical challenges in their acquisition [2]. The use of artificial tooth models in preclinical dental education was first implemented in 1894, and since then, it has grown in popularity to become the most prevalent modality for the simulation of dental procedures in a non‐clinical setting [3]. In most Western institutions, a tooth model or ‘typodont’, generally constructed from a proprietary moulded material, is installed into a ‘phantom head model’, which acts to simulate the patient‐clinician position and management of patient soft tissues. Typodonts constructed using the proprietary material Ivorine (One Dental, Australia and Columbia Dentoform, USA) are amongst the most prevalent models employed by Western dental schools and have been validated in peer‐reviewed publications [4]. The vast majority of typodonts simulate a tooth free from pathology and do not replicate the complex composition of human dentition.

3D printing (also known as additive manufacturing) has emerged as a viable method to fabricate accurate, affordable and complex anatomical models and medical devices [5]. The use of 3D printed dental models in preclinical education has yet to be widely employed in Western dental schools, though preliminary research in this area has demonstrated that both dental students and dentists evaluate them as favourable alternatives and may experience a benefit to the development of technical skills and self‐directed learning [6]. Sinha et al. [7] developed 3D dental models that implemented a lower‐density print region to simulate dental caries and demonstrated that their use improved readiness and reduced stress and anxiety in dental students prior to treating their first caries patient. In the field of prosthodontics, Höhne et al. [8] showed that 3D printed models, which incorporate coloured layers to give real‐time feedback on veneer preparation depth, were perceived as more suitable compared to standard typodonts. Extracted human teeth are still widely utilised in endodontic training, as standard typodonts lack dental pulp and root canal morphology. 3D printed dental models can be constructed with a root canal system that is standardised and appropriately complex for the participant's skill level, thus benefiting learning, improving confidence, and improving formal assessment [9]. There exists vast potential to develop robust and sustainable educational tools for the practical, skills‐based training of dentists.

The basic principle of 3D printing is that a digital model is fabricated layer‐by‐layer, with material selectively deposited to form a 3D object [10]. Numerous methods and materials exist, each with advantages and limitations, making the selection of the correct material paramount [11]. Resin‐based models printed with stereolithography (SLA) printers have been the most extensively described in the literature due to their wide availability, relatively low cost, and high accuracy [7, 12, 13]. Material jetting (MJ) printers possess the ability to fabricate a single structure out of multiple different materials and colours, making them suitable for producing complex models that simulate different tissue types or give feedback on the depth of tooth preparation [14]. Compared to SLA, MJ technology has received less attention in the literature to date, and data comparing it to more widely used technologies and current standard tooth models is lacking. Resin‐based printing technologies have been demonstrated to be superior to FFF and SLS in simulating alveolar bone for dental implant placement [15]; however, whether this applies to the simulation of teeth has not yet been elucidated. Reviews of published literature in this field have demonstrated there to be a lack of studies that incorporate FFF and SLS technologies [16], which may be the only types of 3D printer available at some institutions.

Over the coming decades, the utilisation and understanding of 3D printing technologies are likely to be prominent in dental education, driven by the revolution of clinical practice by chairside indirect restoration systems (CEREC), intra‐oral scanning and the increasing prevalence and affordability of 3D printers [17]. The potential applications and benefits of 3D printed models in preclinical education have been validated, though data that helps inform the application of specific 3D printed technologies and materials has been identified as an important area of future research [6]. Most published studies evaluate a single model without any comparison to models constructed from other 3D printing technologies. A comparison between different technologies is necessary to improve understanding of the opportunities, limitations and best applications of the various 3D printing technologies in dental education. Studies comparing the use of different materials and 3D printing technologies in manufacturing anatomical models have provided valuable results in surgical fields like urology [18] and neurosurgery [19]. The proposed study was designed to address this gap in the literature, which is essential to help educators select the correct 3D printing technology and material to meet the needs of their clinical simulation and help realise the immense promise that 3D printing offers to improve preclinical dental education. Our study aimed to evaluate the qualities of various 3D printed dental training models, utilising a range of different technologies in restorative dental education. To achieve this, a group of dentists and dental students evaluated a range of 3D printed dental models using a Likert scoring system questionnaire. A secondary objective was to determine which features of these models are most important to dentists and dental students.

## 2 Methods

The study involved three stages: (1) model design, (2) fabrication of dental models using five different materials, and (3) evaluation of the models by 12 dental students and six dentists using questionnaires and semi‐structured interviews.

### 2.1 Design

The design of the tooth model was based on a surface scan of current maxillary first molar Ivorine typodonts (One Dental, Australia) used by students at the University of Queensland School of Dentistry. The model was scanned using a Trios 3 intraoral scanner (3Shape, Denmark), with an estimated capture rate of 1875 images per second and a scanning trueness of 20–40 μm [20, 21]. The mesh was fixed and optimised for printing using 3‐Matic CAD software (Materialise, Belgium). Slicing of the CAD model was performed using slicers specific to the print method.

### 2.2 Fabrication

Five different 3D printing technologies were chosen to compare; key properties are summarised in Table 1. Initial dental drilling testing was performed on various materials to reduce the number of samples to 1 material per printing process and avoid survey fatigue on participants. The print time per sample and cost per sample (Table 2) were calculated based on a full print bed, as many print methods have a decrease in cost per unit with increasing units produced. For the SLS print, however, a full print bed would produce over 6000 samples, so a reduced print bed size was used as is customary for smaller print sizes. Print time did not include post‐processing; all processes except FFF have significant post‐processing requirements that can be time‐consuming.

**TABLE 1:** Advantages and disadvantages of 3D printing processes used in this study.

3D printing process	Description	Advantages	Disadvantages
Selective laser sintering (SLS)	The process uses a high‐power laser to selectively fuse small particles of polymer powder into a solid structure, layer by layer	Wide range of materials Large print bed so can have high volume throughput High stiffness and strength material	Printer and post processing equipment can be expensive Single material only Parts can have varied surface texture quality
Fused filament fabrication (FFF)	The process involves heating a thermoplastic filament to a semiliquid state, extruding it through a nozzle, and building layers in a repeating process	Cost effective Simple process and post processing Variety of thermoplastics	Relatively low resolution and accuracy Poor surface quality Requires manual removal of support
Material jetting (MJ)	A printhead deposits micro‐droplets of a photo‐reactive resin onto a build plate, solidifying using ultraviolet (UV) light. It allows for high resolution and complex multi‐material builds, with multiple jets for varying materials	Highest resolution and accuracy of the 3D printing techniques in this study Multi‐material and multi‐colour possible Smooth surface finish	Support can be difficult, and time consuming to remove Significant material wastage during printing Printers and materials can be expensive
Stereolithography (SLA)	A UV light selectively cures liquid photopolymer resin. The build plate moves in and out of the resin vat, creating thin layers for each part	Very high resolution Smooth finish	Single material Cannot vary infill Requires manual removal of support Parts can age and become brittle with UV exposure (e.g., sun)
Digital light processing (DLP)	Very similar to SLA but uses a UV projector to flash the build with a mask to cure the resin	Same as SLA except has the potential for higher build volume	Same as SLA

**TABLE 2:** Overview of sample manufacturing and cost.

3D printing process	3D printer manufacturer	3D printer	Printer price (AUD)	Material supplier	Material name	Material composition	Samples per print	Print time per sample (min)	Material cost per sample (AUD)
SLS	3D Systems	sPro60	800 000	3D Systems	Duraform PA12	Polyamide	192	1.1	0.98
FFF	Ultimaker	S3	8600	Polymaker	PETG, White	Polyethylene terephthalate glycol	99	22	0.06
MJ	Stratasys	J750 DAP	700 000	Stratasys	VeroWhite	Proprietary	512	1.3	1.87
SLA	Formlabs	Form 3	7350	Formlabs	White Resin	Mixer of urethane dimethacrylate and methacrylate monomer(s)	49	6.7	0.57
DLP	Asiga	Pro 4K	60 000	Asiga	DentaModel	Mixer of urethane dimethacrylate and methacrylate monomer(s)	136	1.06	0.3
Ivorine	N/A	N/A	N/A	One Dental	Ivorine		N/A	N/A	N/A

SLS samples were sliced using 3D sprint (3D systems, USA) with a 0.12 mm layer height. Samples were placed in a cage to ensure they could be found after printing. Postprocessing was performed using a Euroblast industrial sandblasting machine (Guyson, United Kingdom).

FFF samples were sliced using Cura slicer (Ultimaker, Netherlands) with a 0.1 mm layer height, 100% infill, 0.8 mm wall thickness and 1.2 mm top/bottom layer thickness.

MJ samples were sliced and automatically oriented using Grabcad (Stratasys, USA) and a layer thickness of 27 μm. Post‐processing was performed using pressure washing as recommended by the supplier.

SLA samples were prepared using PreForm software (Formlabs, USA), with a 0.1 mm layer height and minimal support structures. After 3D printing, samples were washed in 99% isopropyl alcohol for 10 min using Formlabs Form Wash, and cured at 60°C for 60 min, following Formlabs' recommended process for White Resin.

DLP samples were sliced using Asiga slicer (Asiga, Australia), with a 0.05 mm layer height and minimal support structures. After 3D printing, samples were washed in 99% isopropyl alcohol for 5 min in an ultrasonic bath and cured for a total of 4000 flashes in an Otoflash G171 (NK Optik, Germany) following Asiga's recommended process for Dentamodel.

### 2.3 Evaluation

Each sample was randomly fitted into either the right or left first molar space of a maxillary arch dental model (One Dental, Australia) and labelled from A to F. Participants were blinded to the material used to produce each sample. Participants received high and slow‐speed dental handpieces, burs, and a dental mirror (Figure 1).

**FIGURE 1:**
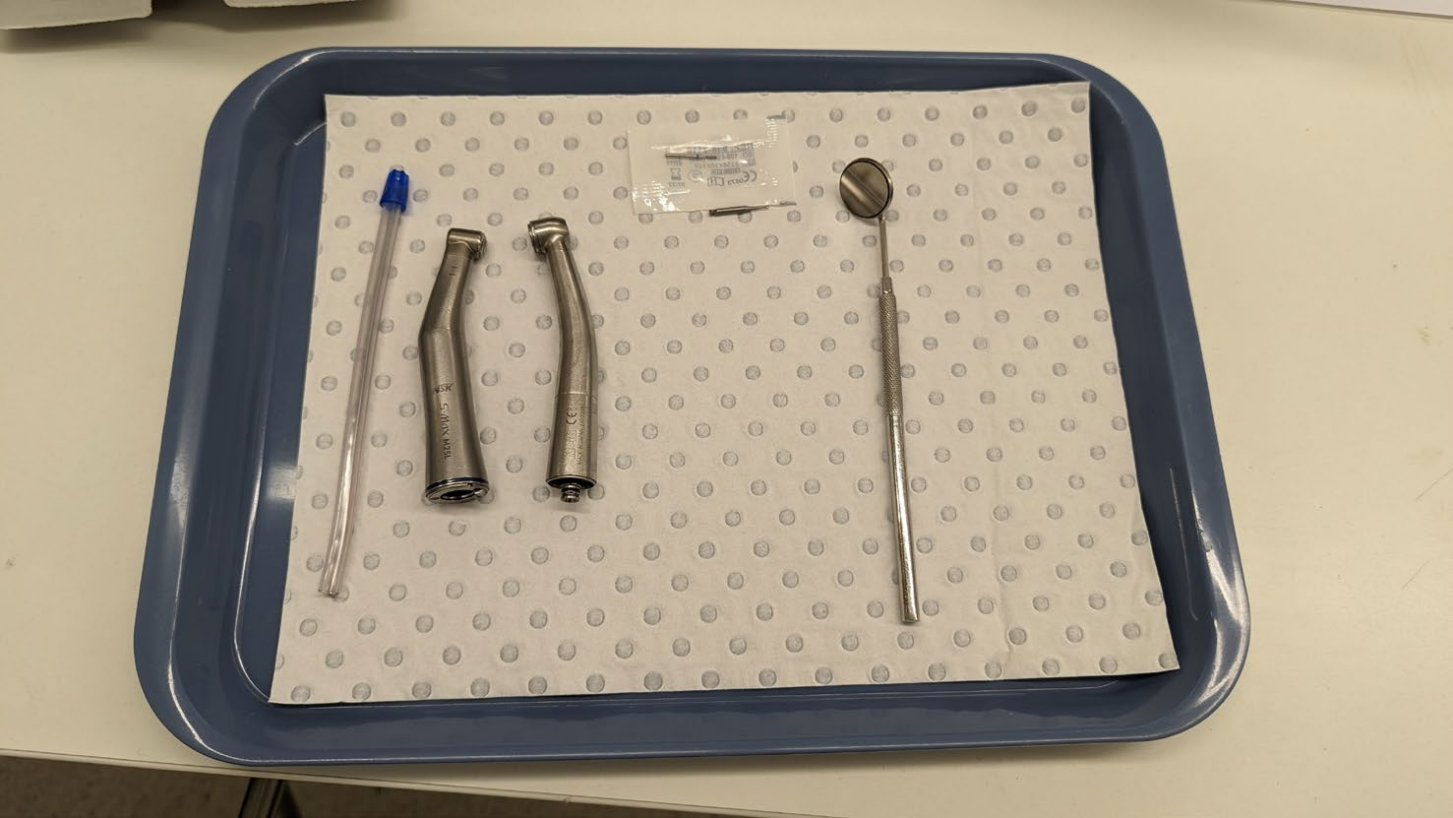
Instruments provided to each participant included a high‐speed handpiece with a flat fissure bur, a slow‐speed handpiece with a round bur, and a dental mirror.

Twelve fourth or fifth‐year dental students and six graduate dentist tutors from the University of Queensland (UQ) attended a session at the UQ Dental Simulation Laboratory (*n* = 18). Participants were given a short survey to evaluate their experience interacting with 3D printed models. They were then asked to rank the qualities they believed most important in creating a 3D printed dental model. Subsequently, participants were asked to place each maxillary arch dental model into the phantom head, perform a class 1 (occlusal) cavity preparation on tooth A and then assess that sample using the questionnaire provided. If desired, participants were allowed to further prepare the tooth after completing the class 1 cavity preparation. Participants were instructed to progress in alphabetical order, and the order of materials was randomised for each participant. The questionnaire collected information about the hepatics in comparison to enamel or dentine, visual appearance, anatomical accuracy and overall suitability as a dental training model on a 5‐point Likert scale from strongly disagree [1] to strongly agree [5].

After completing the simulation, a semi‐structured interview was conducted with each participant individually, facilitated by one of three authors (SB, DC, JN). The facilitator documented responses to four standardised questions to obtain free‐form feedback and further elaborate on the experience.

Statistical analysis was completed using IBM SPSS version 29.2 (IBM Corp., Armonk, New York, USA) software. Dental student and tutor Likert score responses were pooled for analysis and presented in Table 3. Normally distributed data were reported using mean and standard deviation. Statistical differences between groups were calculated using a paired *t*‐test (two‐tailed) with the cutoff for statistical significance set at *p* = 0.05. Bonferroni correction was applied to reduce the familywise error rate given multiple comparison groups. A single author (SB) completed a thematic analysis of the qualitative data. Following the methodology described by Braun and Clark [22], recurrent themes were identified and presented in Table 4.

**TABLE 3:** Mean results for each technology per question with standard deviation (SD) in brackets.

Question	Ivorine	DLP	95% CI	SLA	95% CI	MJ	95% CI	FFF	95% CI	SLS	95% CI	3D printed combined	95% CI
			*p*		*p*		*p*		*p*		*p*		*p*
Haptics enamel	3.67 (1.03)	3.11 (1.08)	−0.07 to 1.18	3 (1.24)	0.03 to 1.31	3.11 (1.41)	−0.15 to 1.26	2.17 (1.2)	0.63 to 2.37	2 (1.13)	0.89 to 2.45	2.68 (1.29)	0.35 to 1.63
			0.38		0.21		0.58		0.01		< 0.001		0.003
Haptics dentine	3.44 (1.10)	3.44 (1.04)	−0.87 to 0.87	3.33 (1.19)	−0.57 to 0.80	3.28 (1.32)	−0.47 to 0.81	1.83 (0.79)	0.85 to 2.38	1.83 (0.99)	0.85 to 2.38	2.74 (1.29)	0.05 to 1.35
			1		1		1		< 0.001		< 0.001		0.003
Visual appearance	4 (0.46)	3.28 (0.96)	0.16 to 1.28	3 (1.09)	0.43 to 1.56	3.39 (1.04)	0.19 to 1.03	1.61 (1.04)	1.79 to 2.98	2.39 (0.98)	1.04 to 2.18	2.73 (1.20)	0.70 to 1.84
			0.07		0.008		0.04		< 0.001		< 0.001		< 0.001
Anatomy	4.28 (0.75)	3.44 (0.98)	0.34 to 1.32	3.33 (0.97)	0.39 to 1.50	3.39 (1.20)	0.19 to 1.59	2.11 (1.13)	1.59 to 2.74	2.5 (1.2)	1.15 to 2.41	2.96 (1.21)	0.74 to 1.91
			0.01		0.01		0.016		< 0.001		< 0.001		< 0.001
Overall suitability	4.17 (0.92)	3.44 (1.34)	−0.04 to 1.48	3.33 (1.24)	0.011 to 1.66	3.33 (1.24)	0.11 to 1.56	1.61 (0.85)	2.07 to 3.04	1.89 (0.96)	1.71 to 2.84	2.72 (1.37)	0.77 to 2.12
			0.30		0.24		0.14		< 0.001		< 0.001		< 0.001

*Note: T*‐test results are all in comparison to Ivorine as the control and 95% confidence interval (CI) and *p* value for each shown.

**TABLE 4:** Themes identified through thematic analysis and a selection of associated responses from participants.

Theme	Standardised question	Selected responses
A more complicated internal structure would be beneficial	What features of a dental training model would you find most beneficial to your education?	‘Layering: enamel, dentine, pulp’ ‘Differentiating dentine and enamel’ ‘If you could simulate decay would be helpful’
The significance of external structure and appearance on dental model performance	What parts of the 3D printed Model do you think needs improving?	‘Accuracy to anatomy not right yet’ ‘Anatomical details. No fissures or guidelines’ ‘Anatomy of DLP needs more definition. Colour, but not critical’
Participants were enthusiastic about the implementation of 3D printing technology in dental training	Is there anything that would prevent you from recommending 3D printing to make educational training models?	‘No. Think it is a good different feel from plastic. Needs to suit procedure’ ‘No. Number 1 was a 3D printed one’ ‘No. Suitable for training. If 3D printed teeth were more available and cheap’

## 3 Results

Table 3 and Figure 2 summarise the pooled responses from all participants after completing an occlusal preparation on each model. SLS and FFF performed significantly inferior to the Ivorine control in all domains. SLA was significantly inferior to Ivorine in all domains apart from dentine haptics. MJ was significantly inferior to Ivorine in all domains apart from enamel and dentine haptics. DLP performed the best and was significantly inferior to Ivorine in visual appearance and anatomy, but not in enamel haptics, dentine haptics, or overall suitability. The pooled results from all 3D printing technologies were rated as significantly inferior to Ivorine in all domains.

**FIGURE 2:**
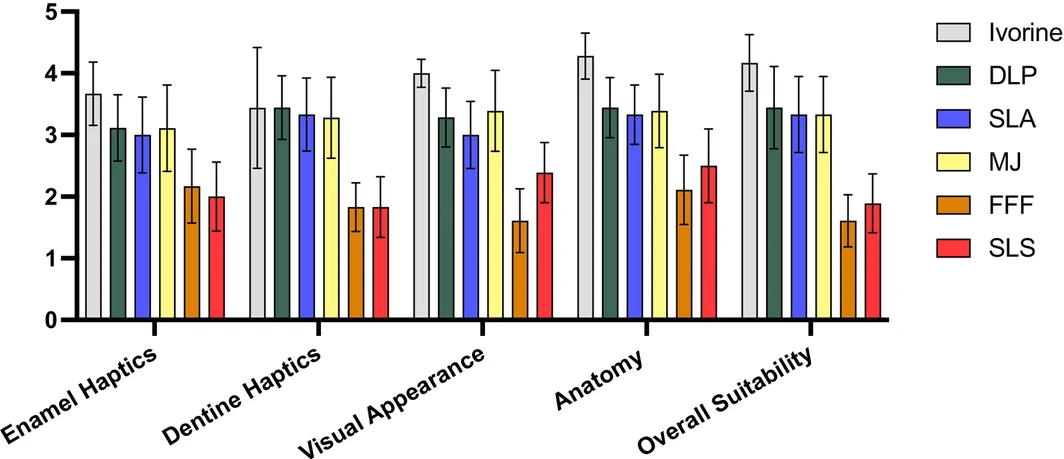
The combined mean response of both dental students and dental tutors (*n* = 18) to 5‐point Likert scale questions for each material, Ivorine (control), DLP (digital light processing), SLA (stereolithography), MJ (material jetting), FFF (fused filament fabrication) and SLS (selective laser sintering). Error bars represent 95% confidence interval (CI).

Previous exposure to 3D printed anatomical models amongst our cohort was low (Figure 3), with 11 dental students reporting 1–5 previous interactions (91.7%) and one student reporting no prior exposure (8.3%). Dental tutors had even less familiarity, with 3 reporting that they had interacted with 3D anatomical models 1–5 times (50%) and 3 reporting no prior exposure (50%).

**FIGURE 3:**
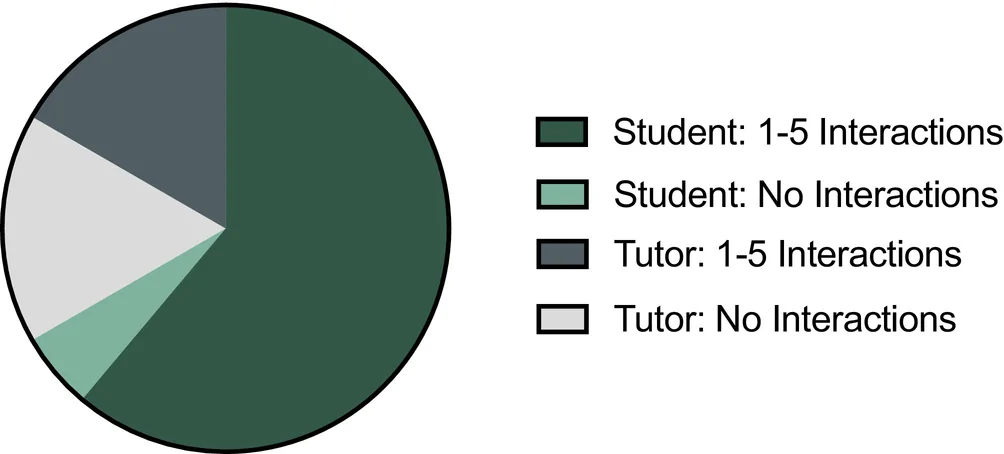
Summary of previous exposure to 3D printed anatomical models amongst participants.

The most important qualities in designing 3D printed dental models were generally ranked similarly by dental students and dental tutors (Figure 4). ‘Realistic feel’ and ‘accurate anatomy’ were overwhelmingly identified as the most important, which correlates with the importance of these features in simulating a realistic experience. Minimising cost was identified as being more important for students, as in most dental schools in Australia, the cost of models is generally the students' responsibility. ‘Quick production’ and ‘realistic appearance’ were generally considered less important to both participant groups.

**FIGURE 4:**
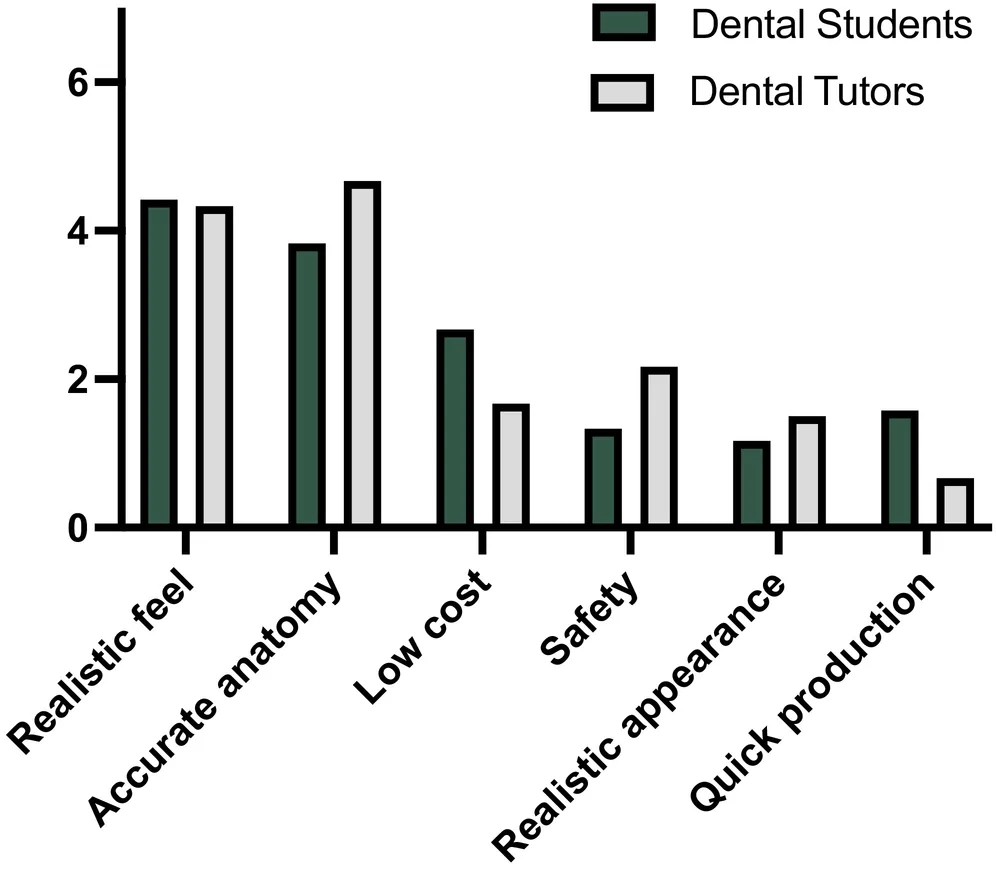
Mean values of responses from dental students (*n* = 12) and dental tutors (*n* = 6) regarding qualities expected from a 3D printed dental training model (6 = highest, 1 = lowest).

Thematic analysis of the semi‐structured interview (Table 4) identified three key themes:

- A more complicated internal structure would be beneficial.
- The significance of external structure and appearance on dental model performance.
- Participants were enthusiastic about the implementation of 3D printing technology in dental training.

## 4 Discussion

3D printing technology has significant potential to benefit dental education, though current standard 3D printing methods and materials still need further development to replace their traditional counterparts. In our study, the DLP model performed best, with no statistically significant difference from Ivorine in terms of enamel haptics, dentine haptics, or overall suitability. DLP was the second most affordable model, had the fastest per‐unit print time, and required very little post‐print processing (Table 2). These findings indicate that DLP is an ideal technology for fabricating dental training models. Surprisingly, there is an absence of published literature assessing the use of DLP models in dental training [6]. This may be partially explained by the greater cost of DLP printers compared to competing SLA and FFF printers.

Further investigation is required to validate our results and confirm if DLP is a superior option to SLA for those with access to a suitable printer.

SLA was the original 3D printing technology, and its use in the fabrication of dental training models is the most described [16]. Concordant with the findings of our study, SLA has been reported to produce models that, while too soft to simulate tooth structure accurately, are otherwise satisfactory [16]. The use of a slow‐speed handpiece or reducing the rotational speed of high‐speed handpieces may be considered to mimic the harder composition of natural teeth, especially enamel; though this will reduce the fidelity of the simulation and is unlikely to completely overcome this limitation. MJ performed similarly to the other resin‐based technologies, being non‐inferior to Ivorine in both haptic domains (Table 3). Unlike other technologies, MJ printers can fabricate models with multiple layers of different materials and colours in a single print, though the high initial cost of the printer, material cost and low material hardness limits the potential for large‐scale utilisation to replace standard typodonts [14]. The successful use of SLA to fabricate multilayer models by printing layers individually has been documented, though this requires significant post‐printing processing [23]. MJ could be used to produce standardised, highly complex models for the simulation of more advanced procedures, such as complex endodontics. For these models, the dentine and pulp could be printed, and ceramic casting used to create the enamel. This strategy would allow for standardised and haptically accurate simulation of highly complex cases, thus justifying the increased cost‐per‐model [24].

The resin‐based technologies (DLP, SLA and MJ) outperformed FFF and SLS in all domains, suggesting that the latter are poorly adapted to fabricate dental training models. FFF was rated as the least suitable model, with semi‐structured interviews revealing that it produced flaky debris when cut, clogging up the cavity and obscuring the cavity margins (Figure 5). This was compounded by poor surface resolution and unrealistic haptic feedback. While the FFF model was difficult to penetrate with a dental bur, it was not in a way that simulated the crystalline structure of enamel. Interestingly, the FFF material (PETG) has been identified as having good potential in fabricating temporal bone models for otolaryngology training [25] and burr hole training in neurosurgery [26]. In the simulation of bone, participants perceived the debris production as beneficial, and it increased simulation fidelity, highlighting the importance of matching the correct material to the simulated clinical scenario. SLS models were similarly reported to be too hard and demonstrated a propensity to melt and adhere to the bur even with irrigation. This feature significantly contributed to its poor performance, as has been previously described [26]. Due to the combination of poor surface texture, unrealistic haptic feedback, and other incompatible properties, resin‐based technologies should be favoured over FFF and SLS in the production of dental models.

**FIGURE 5:**
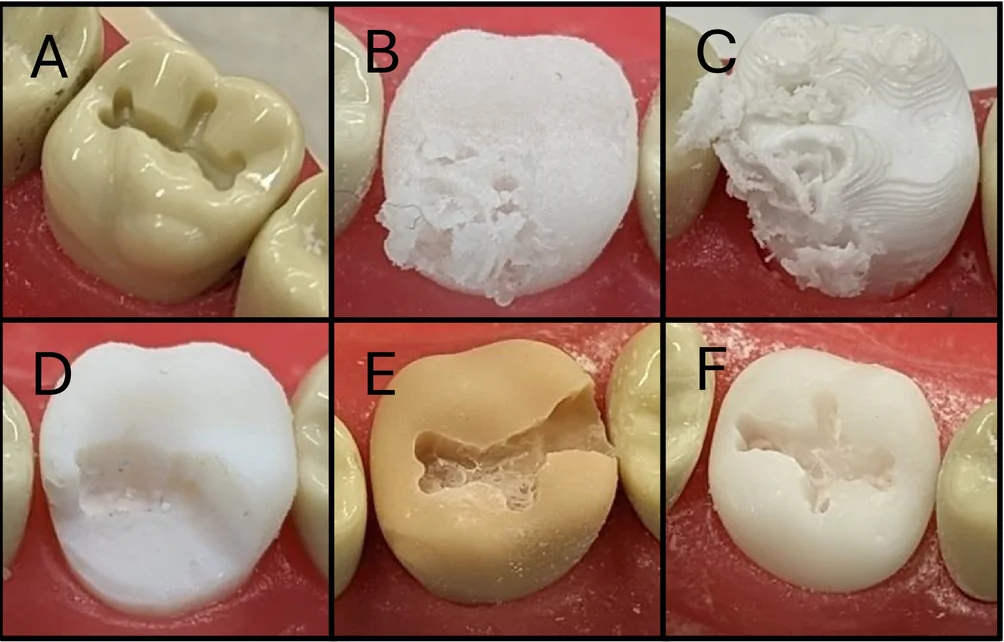
Close‐up photographs taken of tooth models after cavity preparation. Ivorine (A), selective laser sintering (B), fused filament fabrication (C), material jetting (D), digital light processing (E), stereolithography (F).

Participants generally reported limited prior exposure to 3D printed models. However, students reported more experience with 3D printed models, signifying an increasing penetration of digital dentistry in the curriculum of Australian dental schools. ‘Realistic feel’ and ‘accurate anatomy’ were rated as the most important features of dental models by both students and tutors. This is unsurprising given the importance of haptic feedback in restorative dental procedures and the significance of anatomy for both cavity preparation design and accurate restoration. An important feature for students was ‘low cost’, highlighting the financial pressure dental education places on students as it is primarily their responsibility to pay for education resources. The production of training models that provide acceptable education value at a reduced cost will improve access to dental training and alleviate the impact of financial concerns associated with the significant stress and mental health problems experienced by dental students [27].

The thematic analysis revealed further insights into participants' perception of 3D printed dental training models and what the goals should be for improving the utility of this technology (Table 4). Theme 1: A more sophisticated internal structure of models may improve their utility, with participants particularly desiring differentiation between enamel, dentine and pulp. Early attempts to produce internally complicated models have revealed the potential for 3D printing technology to eventually outperform standard methods in this domain. SLA technology has been used to create multicoloured models that provide feedback on crown preparation depth [23] and has been favourably assessed in simulating direct pulp capping [28]. Widespread application of these models may be challenging as both required significant post‐print processing, including injecting a polyether dental impression material to simulate pulpal tissue. MJ technology offers the ability to produce highly complex multi‐material models that will allow for varying haptic changes throughout the simulation [14]. Theme 2: The surface appearance and structure of the 3D printed models were poorly perceived by participants. The lack of more defined occlusal anatomy was attributable to the resolution limitations of the post‐scan processing, as many of the printers used in this study can print in a resolution sufficient to replicate fine details. High‐resolution scanning devices and careful processing should be utilised in the production of future models to produce a smooth surface and well‐defined occlusal anatomy. Material colour was not described as having a high impact on the overall performance of the models, which is supported by our data demonstrating that the appearance of dental training models is of relatively low importance compared with other features (Figure 4). Theme 3: Participants reported an overwhelmingly positive desire to interact with 3D printed models in the future and tended to support their implementation in the pre‐clinical training. Students valued the ability to interact with a wide variety of learning tools. They were equally interested in understanding how to incorporate digital and CADCAM technologies into their learning and future clinical practice. 3D printed teeth offer the ability to recreate exact patient anatomy, allowing a close simulation of a procedure before completing it clinically, improving patient outcomes and clinician confidence [29]. The increasing adoption of this technology in clinical practice is likely to improve access and decrease upfront costs associated with its use in the education sector.

Our models were produced at a state‐of‐the‐art facility with access to various current‐generation 3D printers. This is unrealistic for all dental schools to access, with most having limited 3D printer options, and thus, our results may not be generalisable to other institutions. Our results should still prove valuable to education providers seeking to decide which 3D printing technology will best suit the needs of their institution. The study was conducted at a single centre, which may affect the generalisability of our results, though we have included both undergraduate students and qualified dentists. The relatively small sample size is comparable to similar studies [7] and the presence of multiple significant findings is reassuring that the sample size is reasonable for the degree of differences between the groups. The financial analysis displayed in Table 2 does not consider the cost associated with printer maintenance and operation, which in some cases can be substantial. Participants were only tasked to complete a simple occlusal cavity preparation, and the models were not assessed for performance in simulating tooth restoration and more complicated dental procedures. The subjective nature of our data would benefit from further validation via more complicated objective testing and assessment of the mechanical properties of each material.

This study is the first to compare different materials on their suitability in constructing 3D printed dental models, and the results can be utilised to inform educators on the strengths and limitations of the most common 3D printing technologies. Educators should focus on using resin‐based technologies, with DLP and SLA being the most cost‐effective, while MJ allows the most freedom and complexity in model design. Where financial and technical limitations allow, emphasis should be given to constructing models with realistic haptic feedback, accurate external anatomy and appearance, and the incorporation of internal anatomical features such as distinct enamel, dentine and pulpal layers. The contemporary incorporation of 3D printing technology into the dental curriculum should focus on supplementing the limitations of existing typodonts, such as restricted complexity and internal structure. Future studies may seek to incorporate the objective testing of mechanical properties to understand how a 3D printed dental model's response to tensile, compressive, shear and torsional forces affects its suitability as a simulation material and haptic feedback. Studies that include other types of access cavity preparations, such as interproximal or cervical lesions, and the simulation of direct composite restoration would further inform educators on material selection. The use of 3D printed models in the education of oral surgery and orthodontics has received less attention than other dental fields, and studies designed to address this gap would clarify if 3D printed models can help clinicians improve skills specific to these areas.

## 5 Conclusion

This study assessed the qualities of five unique 3D printed dental models in comparison to standard Ivorine typodonts. MJ models were by far the most expensive per unit to produce, being over three times as expensive as the other resin‐based technologies (SLA and DLP). FFF offers a more affordable alternative, though it is limited by a high per‐unit production time and many unfavourable characteristics for dental simulation. The visual appearance and anatomy of the Ivorine model were significantly better than all other models (*p* < 0.05). Resin‐based technologies (DLP, SLA and MJ) were not significantly inferior to Ivorine in the domains of haptic feedback and overall suitability (*p* > 0.05). When considering both printing and material characteristics, DLP offers cost‐effective models with acceptable performance in restorative dental procedure simulations. Further work is needed to develop 3D printable materials that perfectly mimic dental tissue. This study also utilised surveys and interview questions to better understand which features of dental models are most important to users and assess perception around the incorporation of 3D printed technologies in dental education. Participants valued models that incorporated a realistic feel, accurate anatomy and the inclusion of internal features to simulate enamel, dentine and pulp layers and demonstrated a positive attitude towards the incorporation of 3D printed models in their education. Collectively, the results of this study should benefit educators in making material selection and model design decisions to fabricate economically viable models that complement their clinical simulation. The well‐informed incorporation of this technology into dental education is essential to producing technologically literate clinicians and supporting the development of fundamental clinical skills. 3D printed models may supplement the limitations of standard Ivorine models, improving self‐education and visual feedback, allowing practice on patient‐specific models and facilitating the simulation of complex procedures such as endodontics.

## Funding

The authors have nothing to report. Materials were provided by the Herston Biofabrication Institute, and facilities were provided by the University of Queensland.

## Ethics Statement

This study was approved by the Human Research Ethics Committee at The University of Queensland (2022/HE001251). The data supporting this study's findings is not shared publicly due to privacy and ethical restrictions. There are no conflicts of interest to declare.

## Conflicts of Interest

The authors declare no conflicts of interest.

## Data Availability

The data that support the findings of this study are available on request from the corresponding author. The data are not publicly available due to privacy or ethical restrictions.
